# Analysis of mechanical ventilation and lipopolysaccharide-induced acute lung injury using DNA microarray analysis

**DOI:** 10.3892/mmr.2015.3335

**Published:** 2015-02-11

**Authors:** YUQING CHEN, XIN ZHOU, LING RONG

**Affiliations:** 1 Department of Respiratory Medicine, Shanghai First People’s Hospital, Shanghai Jiaotong University School of Medicine, Shanghai 200080, P.R. China; 2 Department of Respiratory Medicine, The People’s Hospital of Bozhou, Bozhou, Anhui 236804, P.R. China

**Keywords:** acute lung injury, mechanical ventilation, lipopolysaccharide, hierarchical clustering, functional enrichment analysis, pathway enrichment analysis

## Abstract

Gene expression profiles of samples taken from patients with acute lung injury (ALI) induced by mechanical ventilation (MV) and lipopolysaccharide (LPS) were analyzed in order to identify key genes, and explore the underlying mechanisms. The GSE2411 microarray data set was downloaded from the Gene Expression Omnibus. This data set contained microarray data from 24 mouse lung samples, which were equally divided into four groups: Control group, MV group, LPS group and MV+LPS group. Differentially expressed genes (DEGs) were identified in the MV, LPS and MV+LPS groups, as compared with the control group, using packages of *R* software. Hierarchical clustering and between-group comparisons were performed for each group of DEGs. Overrepresented biological processes were revealed by functional enrichment analysis using the Database for Annotation, Visualization and Integrated Discovery. Unique DEGs in the LPS and MV+LPS groups were selected, and pathway enrichment analyses were performed using the Kyoto Encyclopedia of Genes and Genomes Orthology Based Annotation system. A total of 32, 264 and 685 DEGs were identified in the MV, LPS and MV+LPS groups, respectively. The MV+LPS group had more DEGs, as compared with the other two treatment groups. Genes associated with the immune and inflammatory responses were significantly overrepresented in both the LPS and MV+LPS groups, suggesting that LPS dominated the progression of ALI. Unique DEGs in the LPS and MV+LPS groups were associated with cytokine-cytokine receptor interaction. The Janus kinase-signal transducer and activator of transcription signaling pathway was shown to be enriched in the LPS+MV-unique DEGs. The results of the present study demonstrated that MV could exaggerate the transcriptional response of the lungs to LPS. Numerous key genes were identified, which may advance knowledge regarding the pathogenesis of ALI.

## Introduction

Acute lung injury (ALI) is a clinical syndrome associated with respiratory dysfunction, which is caused by numerous local or systemic inflammatory stimuli. ALI is predominantly caused by sepsis, and the rate of ALI-associated mortality is 50-60% (1).

Considerable experimental and clinical evidence has demonstrated that pro- and anti-inflammatory cytokines exhibit a major role in the pathogenesis of inflammatory-induced ALI (2-4). Numerous key genes and potential treatments for ALI have been proposed. Abraham *et al* (5) previously reported that high mobility group protein-1 is a distal mediator of inflammatory ALI. Furthermore, Imai *et al* (6) indicated that the Toll-like receptor 4 signaling pathway has a key role in ALI. Mei *et al* (7) demonstrated that mesenchymal stem cells overexpressing angiopoietin 1 were able to prevent lipopolysaccharide (LPS)-induced ALI in mice. In addition, caspase inhibitor (8), urinary trypsin inhibitor (9) and sphingosine 1-phosphate (10) have been shown to exhibit protective functions in LPS-induced ALI.

LPS is a major bioactive component of endotoxin, which can lead to sepsis and ALI (11). Rittirsch *et al* (12) previously reported that the migration inhibitory factor and leukotriene B_4_ mediator pathways are involved in the immunopathogenesis of LPS-induced experimental ALI; however, complement activation did not contribute to the development of ALI in an LPS model.

For patients with severely impaired respiratory function, mechanical ventilation (MV) is an essential means of treatment (13,14). MV provides effective respiratory support; however, it may also result in severe lung damage (15,16). Ranieri *et al* (17) previously verified that MV is able to induce a cytokine response, which may be attenuated by therapeutic strategies that minimize overdistention and recruitment/derecruitment of the lung. Therefore, determining the similarities and differences between MV- and LPS-induced ALI is required in order to produce more effective and safe treatment.

In the present study, gene expression profiles of mice with ALI induced by MV, LPS and MV+LPS were compared with controls, in order to identify differentially expressed genes (DEGs). Unique DEGs for each treatment group were screened, and the differences in the induction mechanisms between the groups were discussed.

## Materials and methods

### Gene expression data

The GSE2411 microarray data set (18) was downloaded from the Gene Expression Omnibus (19). Raw data were collected from 24 C57/B6 male mice, which were randomly divided into four groups: Control, phosphate-buffered saline (PBS) aspiration without MV; MV treatment, PBS with MV; LPS treatment, LPS aspiration without MV; and MV+LPS treatment. The annotation information for the chip was downloaded from the Mouse Expression 430A Array (GPL339 platform; Affymetrix, Santa Clara, CA, USA).

### Data pre-treatment and differential analysis

The probes were mapped to genes according to the annotation files. When numerous probes were mapped to a single gene the values were averaged, in order to obtain the final expression levels. Log_2_ transformation and quantile normalization of the gene expression levels were conducted using the affy package (20) of *R* software (http://www.R-project.org). The three experimental groups were compared with the control group: Control vs. MV group; control vs. LPS group; and control vs. MV+LPS group. Statistical analyses were conducted using the multtest package (21) of *R* software, and numerous testing corrections were conducted using the Benjamin-Hochberg method (22). A false discovery rate (FDR)<0.05 and |logFC|>1 were set as the cut-off values, in order to screen for DEGs.

### Cluster analysis

Hierarchical clustering (23) was performed for the DEGs identified from the three groups using *cluster* 3.0 program (version 1.47; http://rana.lbl.gov/EisenSoftware.html). The results were then visualized using Tree view program (http://rana.lbl.gov/EisenSoftware.html) (23).

### Between-group comparisons of the DEGs

Student’s t-test (24) was used to examine the differences in the gene expression levels of the DEGs between the three experimental groups. P<0.05 was considered to indicate a statistically significant difference, and was used as a threshold value.

### Functional enrichment analysis

To identify the altered biological functions following the various treatments, a functional enrichment analysis was conducted for the DEGs using the Database for Annotation, Visualization and Integrated Discovery (DAVID; http://www.david.niaid.nih.gov) (25). An FDR<0.05 was set as the cut-off value.

### Screening of unique DEGs

The three experimental groups of DEGs were compared, in order to screen for unique DEGs in each experimental group (MV, LPS and MV+LPS groups). The unique DEGs were used to identify the molecular mechanism of ALI, induced by the corresponding treatment.

### Pathway enrichment analysis of the unique DEGs

A pathway enrichment analysis of the unique DEGs was conducted using the Kyoto Encyclopedia of Genes and Genomes Orthology Based Annotation system (KOBAS; HTTP://KOBAS.CBI.PKU.EDU.CN) (26–27). An FDR<0.05 was set as the cut-off value.

## Results

### DEGs

**Figure 1:**
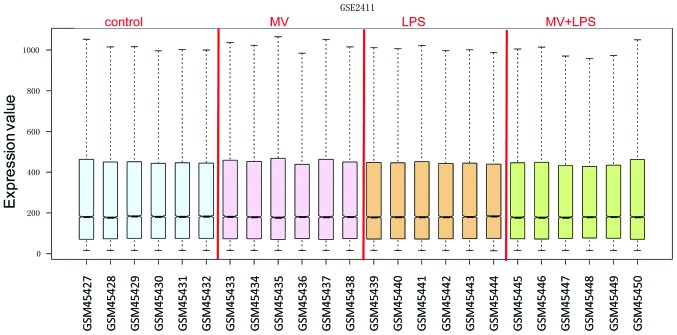
Box plot for normalized gene expression data. From left to right: Control, MV, LPS and MV+LPS-induced acute lung injury samples. The black lines in the boxes represent the medians, which were almost identical, indicating a good level of standardization. MV, mechanical ventilation; LPS, lipopolysaccharide.

The normalized gene expression data are presented in Fig. 1. A total of 32, 264 and 685 DEGs were identified in the MV, LPS and MV+LPS groups, as compared with the control group, respectively. Transcriptional response was detected in each group. Treatment with MV generated the smallest number of DEGs, followed by LPS treatment. Simultaneous application of the two treatments resulted in a significant increase in the transcriptional response.

### Cluster analysis results

**Figure 2:**
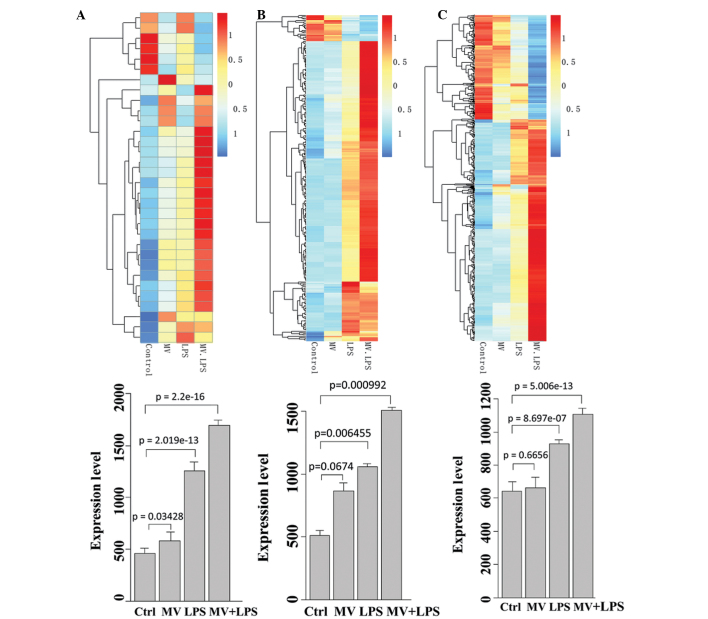
Heat maps for the expression levels of DEGs across all 24 samples (upper), and comparison charts for the expression levels of the DEGs between the four groups (lower). (A) DEGs in the MV group, as compared with the control group. (B) DEGs in the LPS group, as compared with the control group. (C) DEGs in the MV+LPS group, as compared with the control group. DEGs, differentially expressed genes; Ctrl, control; MV, mechanical ventilation; LPS, lipopolysaccharide.

The cluster analysis results are shown in the upper section of Fig. 2. Each group of DEGs could be used to distinguish the corresponding experimental group from the control group. The MV group (Fig. 2A) had fewer DEGs, as compared with the LPS and MV+LPS groups (Fig. 2B and C).

### Results of between-group comparisons

The expression levels of the DEGs in each experimental group were compared using a Student’s t-test (24). The results are presented in the lower section of Fig. 2. The MV+LPS group exhibited the highest degree of overexpression, as compared with the other groups. This result is concordant with the cluster analysis. A combination of MV and LPS stimulated the strongest transcriptional response in the lungs of the mice.

### Functional enrichment analysis results

A Gene Ontology enrichment analysis was performed using DAVID, and the top 10 terms that met the threshold value (P<0.05) are presented in Table I. Negative regulation of catalytic activity was the most significantly enriched term in the MV group. Whereas, immune response was the most significantly enriched term in the LPS and MV+LPS groups, suggesting that LPS dominated the stimulation process of ALI. LPS increased the expression of genes associated with the immune and inflammatory response, whereas MV aggravated this effect.

### Unique DEGs in each experimental group

**Figure 3:**
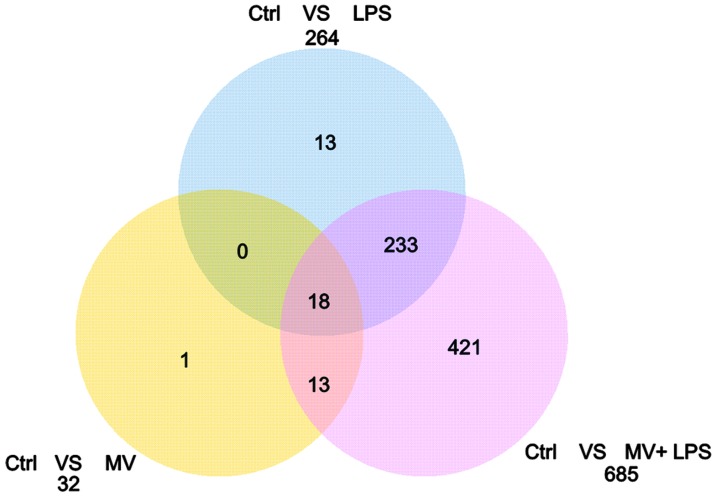
Venn diagram of overlapping differentially expressed genes identified in the three experimental groups. Ctrl, control; MV, mechanical ventilation; LPS, lipopolysaccharide.

The three groups of DEGs were compared, in order to identify unique DEGs in each experimental group. A total of 421 unique DEGs were identified in the MV+LPS group, as compared with MV and LPS groups, whereas 246 unique DEGs were identified in the LPS group, as compared with the MV group (Fig. 3).

### Pathway enrichment analysis results

A pathway enrichment analysis was performed for the two groups of unique DEGs (LPS-unique and MV+LPS-unique), using KOBAS. The overrepresented pathways are presented in Table II. Cytokine-cytokine receptor interaction was the most significantly enriched term in both groups of unique DEGs. The Janus kinase-signal transducer and activator of transcription (Jak-STAT) signaling pathway was enriched for in the LPS+MV-unique DEGs.

## Discussion

The present study identified 32, 264 and 685 DEGs in the MV, LPS, and MV+LPS groups, respectively. The transcriptional response of the lungs was observed in the MV and LPS treatment groups; however, the MV+LPS generated the most DEGs, thus suggesting that MV is able to augment the influence of LPS on gene expression. MV exerted a smaller disturbance on the transcription of genes in the lungs, as compared with LPS, which was further verified by a cluster analysis and between-group comparisons. The underlying mechanism of the effects of MV was obviously different from that of LPS. Negative regulation of catalytic activity was the most significant biological process enriched for in the DEGs of MV, whereas the immune and inflammatory response were the most significantly enriched terms in the DEGs of the LPS and MV+LPS groups.

In order to further determine the differences between the two treatments in causing ALI, the identified DEGs from each experimental group were compared. A total of 246 out of the 264 identified DEGs were unique to the LPS group, as compared with the MV group. A total of 421 novel DEGs were identified in the MV+LPS group, as compared with the MV and LPS groups. Cytokine-cytokine receptor interaction was the most significantly enriched pathway in the LPS-unique and MV+LPS-unique DEGs, which verified the role of the inflammatory response in the pathogenesis of ALI. This result also suggested that MV could exaggerate the degree of inflammation in the lungs, as more cytokines were dysregulated.

Cytokines are synthesized and secreted by inflammatory cells, and have a wide range of biological activities. Cytokines are involved in the regulation of immune cell differentiation and development, and the immune and inflammatory response (28). They are considered to have important roles in ALI (29). In the present study, members of the CXC subfamily, CC subfamily, tumor necrosis factor (TNF)-receptor superfamily and interleukin (IL)-1 cytokine family were shown to be differentially expressed. Chemokine (C-X-C motif) ligand 1 (CXCL1) has previously been shown to have a role in inflammation, and functions as a chemoattractant for neutrophils (30,31). In addition, IL-6 is secreted into the serum, and induces a transcriptional inflammatory response through IL-6 receptor α (32,33). Ahuja *et al* (34) previously reported that circulating IL-6 mediates lung injury, via CXCL1 production following acute kidney injury in mice. IL-1β is a family of proinflammatory cytokines, which are hypothesized to be involved in numerous acute and chronic diseases (35). Kolb *et al* (36) previously demonstrated that transient expression of IL-1β was able to induce ALI. Furthermore, CCL2 is involved in inflammatory disorders of the lung, and it has been suggested that it may contribute to ALI (37). Elevated plasma levels of soluble TNF receptors are associated with morbidity and mortality in patients with ALI (38). Furthermore, numerous members of the colony stimulating factors (CSFs) were identified in ALI in the present study, such as CSF1, CSF2 and CSF3. CSFs have been shown to exhibit protective effects on ALI (39-41). These results suggest that the findings of the present study may be useful in guiding the further investigation of ALI.

The Jak-STAT signaling pathway was shown to be enriched in the MV+LPS-unique DEGs. The Jak-STAT pathway participates in transcriptional activation in response to interferons, and other extracellular signaling proteins (42). It is important in regulating cytokine-dependent gene expression, and cellular development and survival (43). Previous studies have demonstrated that the STAT pathway is activated in ALI (44-45). Severgnini *et al* (46) previously showed that inhibition of Src and Jak kinases resulted in protection against LPS-induced ALI. Therefore, the present study hypothesized that DEGs associated with this pathway may be potential therapeutic targets. CSF3 is a cytokine that controls the production, differentiation, and function of granulocytes (47). Hierholzer *et al* (48) reported that the presence of G-CSF alone in the lung can lead to the recruitment of neutrophils, lung injury, and impaired pulmonary function, thus suggesting that the local production of G-CSF may contribute to the development of lung damage and possibly ALI. Furthermore, Suratt *et al* (49) showed that plasma levels of G-CSF correlate with clinical outcomes in patients with ALI. Pim kinases are a family of serine/threonine kinases whose activity can be induced by allergy-associated cytokines (50). Shin *et al* (50) previously reported that inhibition of Pim1 kinase activation attenuated allergen-induced airway hyperresponsiveness and inflammation. Therefore, Pim1 may exert similar functions in ALI and could be a potential therapeutic target.

Fatty acid metabolism was also found to be significantly enriched for in the MV+LPS-unique DEGs. Murray *et al* (51) demonstrated that select dietary fatty acids are able to attenuate cardiopulmonary dysfunction, during ALI in pigs. However, Rice *et al* (52) reported that twice-daily enteral supplementation of omega-3 fatty acids, γ-linolenic acid and antioxidants did not improve the primary end-point of ventilator-free days, or other clinical outcomes in patients with ALI, and may be harmful. Therefore, although certain findings have been obtained, the association between fatty acid metabolism and ALI remains under debate.

In conclusion, the present study discussed the similarities and differences between MV- and LPS-induced ALI. These findings not only supplement the current knowledge regarding ALI, but may also provide potential biomarkers for the diagnosis and treatment of ALI.

## Figures and Tables

**Table I:** Overrepresented biological processes in each group of differentially expressed genes.

A, Control-MV		
Term	Count	FDR
GO:0043086~negative regulation of catalytic activity	5	3.23E-05
GO:0044092~negative regulation of molecular function	5	9.19E-05
GO:0030595~leukocyte chemotaxis	2	4.86E-02
GO:0060326~cell chemotaxis	2	4.86E-02

B, Control-LPS		

Term	Count	FDR

GO:0006955~immune response	60	5.94E-34
GO:0006952~defense response	51	1.07E-25
GO:0009611~response to wounding	45	1.62E-24
GO:0006954~inflammatory response	38	3.02E-24
GO:0007610~behavior	28	3.39E-07
GO:0007626~locomotory behavior	27	9.83E-12
GO:0042127~regulation of cell proliferation	27	5.45E-04
GO:0006935~chemotaxis	26	2.59E-19
GO:0042330~taxis	26	2.59E-19
GO:0010033~response to organic substance	23	2.44E-02

C, Control-MV+LPS		

Term	Count	FDR

GO:0006955~immune response	87	3.50E-32
GO:0009611~response to wounding	68	7.48E-26
GO:0006954~inflammatory response	54	2.80E-24
GO:0006952~defense response	70	1.19E-20
GO:0042330~taxis	32	7.08E-16
GO:0006935~chemotaxis	32	7.08E-16
GO:0042127~regulation of cell proliferation	62	2.63E-11
GO:0007626~locomotory behavior	34	2.40E-07
GO:0001775~cell activation	33	2.13E-06
GO:0001817~regulation of cytokine production	24	4.65E-06

MV, mechanical ventilation; LPS, lipopolysaccharide; Count, number of enriched differentially expressed genes; FDR, false discovery rate.

**Table II:** Overrepresented pathways in the two groups of unique differentially expressed genes.

A, LPS-unique			
Pathway ID	Pathway name	Count	FDR
mmu04060	Cytokine-cytokine receptor interaction	30	2.44E-13
mmu04062	Chemokine signaling pathway	19	4.93E-06
mmu04621	NOD-like receptor signaling pathway	12	1.42E-05
mmu04620	Toll-like receptor signaling pathway	13	2.48E-04

B, LPS+MV-unique			

Pathway ID	Pathway name	Count	FDR

mmu04060	Cytokine-cytokine receptor interaction	22	1.62E-05
mmu04062	Chemokine signaling pathway	14	3.88E-03
mmu04630	Jak-STAT signaling pathway	12	7.01E-03
mmu04620	Toll-like receptor signaling pathway	9	1.10E-02
mmu00760	Nicotinate and nicotinamide metabolism	4	4.03E-02
mmu00071	Fatty acid metabolism	5	4.89E-02
mmu01040	Biosynthesis of unsaturated fatty acids	4	4.91E-02

LPS, lipopolysaccharide; MV, mechanical ventilation; Count, number of enriched differentially expressed gene; FDR, false discovery rate.
